# Comparative prognostic performance of quick sequential organ failure assessment, lactate, systemic inflammatory response syndrome criteria, and neutrophil-to-lymphocyte ratio for mortality prediction in critically ill dogs and cats

**DOI:** 10.14202/vetworld.2026.2208-2220

**Published:** 2026-05-27

**Authors:** Burin Boonsri, Thanatcha Chaengcheroen, Jidapa Jitprasert, Charisa Suriyawong, Pinkarn Chantawong

**Affiliations:** 1 Faculty of Veterinary Medicine, Chiang Mai University, Chiang Mai 50100, Thailand; 2 Research Center of Producing and Development of Products and Innovations for Animal Health and Production, Faculty of Veterinary Medicine, Chiang Mai University, Chiang Mai 50100, Thailand

**Keywords:** cats, critical-care, dogs, lactate, mortality prediction, neutrophil-to-lymphocyte ratio, qSOFA, systemic inflammatory response syndrome

## Abstract

**Background and Aim:**

Early identification of critically ill dogs and cats at high-risk of mortality is essential for timely intervention and improved clinical outcomes in veterinary emergency and critical-care practice. Several prognostic indicators, including the quick sequential organ failure assessment (qSOFA), blood lactate concentration, systemic inflammatory response syndrome (SIRS) criteria, and neutrophil-to-lymphocyte ratio (NLR), have been proposed for risk-stratification; however, comparative evidence in mixed populations of critically ill small animals remains limited. This study aimed to compare the prognostic performance of qSOFA, lactate, SIRS, and NLR for predicting mortality in critically ill dogs and cats admitted to a critical-care unit (CCU).

**Materials and Methods:**

A prospective cohort study was conducted on 76 client-owned critically ill animals, including 39 dogs and 37 cats, admitted to the CCU of the Small Animal Hospital, Chiang Mai University, Thailand. Admission qSOFA scores, blood lactate concentrations, SIRS criteria, and NLR values were recorded. Survival status at 21 days post-admission was used as the primary outcome measure. Prognostic performance was assessed using receiver operating characteristic curve analysis, area under the curve (AUC), sensitivity, and specificity at clinically relevant cut-offs.

**Results:**

The overall 21-day survival rate was 47.4%, with survival rates of 51.3% in dogs and 43.2% in cats. qSOFA demonstrated the best prognostic performance for mortality prediction, with an AUC of 0.72 (95% confidence interval [CI]: 0.61–0.82). Animals with qSOFA scores ≥ 2 showed significantly higher mortality risk. The combined qSOFA+lactate model produced a slightly higher AUC of 0.75 (95% CI: 0.62–0.88), although the improvement was not statistically significant. Lactate thresholds showed high sensitivity but poor specificity, particularly at lower cut-offs. SIRS and NLR exhibited limited discriminative ability and poor overall predictive performance. Among evaluated triage rules, qSOFA ≥ 2 provided the most balanced sensitivity and specificity for identifying non-survivors.

**Conclusion:**

qSOFA was the most reliable and clinically practical prognostic indicator among the evaluated parameters in critically ill dogs and cats. Lactate may serve as an adjunctive escalation marker for closer monitoring, whereas SIRS and NLR showed limited prognostic utility. These findings support the integration of qSOFA into evidence-based triage and risk-stratification protocols in veterinary critical-care.

## INTRODUCTION

Critically ill dogs and cats represent a heterogeneous group of emergency presentations, including sepsis, shock, trauma, neurologic or obstetric emergencies, and other severe disorders. These conditions may or may not primarily involve systemic hypoperfusion. Regardless of the underlying pathology, critically ill patients frequently experience profound physiologic disturbances and are at increased risk of complications, multiple organ dysfunction, prolonged hospitalization, and death. Early recognition of patients at high-risk of clinical deterioration is therefore essential to optimize monitoring intensity, guide therapeutic decision-making, initiate timely intervention, and improve survival outcomes [1–4].

In both human and veterinary medicine, several scoring systems and biomarkers have been developed to evaluate disease severity and predict clinical outcomes. These include disease-specific indices, such as the Canine Acute Pancreatitis Severity score [5–7], the Animal Trauma Triage score [8–11], and the Modified Glasgow Coma Scale [8, 12], as well as diagnosis-independent prognostic systems, including the neutrophil-to-lymphocyte ratio (NLR) [13], the Acute Patient Physiologic and Laboratory Evaluation full and fast scores [14–16], the systemic inflammatory response syndrome (SIRS) criteria [2, 17, 18], and the Sequential Organ Failure Assessment (SOFA) score [1, 2, 4]. Among these systems, the SIRS criteria have been widely used to identify systemic inflammation and sepsis in dogs and cats, serving as an early screening approach for critically ill patients [18, 19]. SIRS may result from numerous infectious and non-infectious conditions, including sepsis, trauma, pancreatitis, immune-mediated disorders, neoplasia, and postoperative inflammation. However, despite its widespread use, the SIRS criteria have been criticized for poor specificity [20] and limited prognostic accuracy for mortality prediction [18, 21], because similar inflammatory responses may occur across diverse disease processes and varying severities of illness.

To address these limitations, the Sepsis-3 consensus definitions introduced the quick sequential organ failure assessment (qSOFA) score as a simplified bedside tool to identify patients at increased risk of poor clinical outcomes [22, 23]. The qSOFA score incorporates three readily obtainable clinical variables: altered mentation, hypotension (systolic blood pressure ≤100 mmHg), and tachypnea (respiratory rate ≥22 breaths/min). Each variable is assigned one point, allowing rapid and practical risk-stratification in emergency and critical-care settings. Although qSOFA is associated with organ dysfunction, it does not represent a comprehensive assessment of organ failure, which remains the primary function of the SOFA score. In human medicine, a qSOFA score ≥2 has been associated with increased mortality risk, prolonged hospitalization, and longer critical-care unit (CCU) stay among patients with sepsis and critical illness [24].

The NLR is another easily obtainable biomarker derived from routine hematologic analysis and reflects the balance between innate immune responses mediated by neutrophils and adaptive immune responses mediated by lymphocytes. Increased NLR values are thought to result from stress-associated neutrophilia and lymphopenia induced by systemic inflammation and activation of the hypothalamic-pituitary-adrenal axis. In veterinary medicine, NLR has been investigated as a prognostic and inflammatory marker in conditions such as trauma, sepsis, inflammatory diseases, and cardiac disorders, although its clinical utility and predictive consistency remain variable across studies [13, 25].

Recent veterinary investigations have evaluated qSOFA in dogs and cats, demonstrating its potential value as a simple triage and prognostic tool [1, 2]. However, most available studies have focused primarily on disease-specific populations, including pyometra, sepsis, and surgically treated peritonitis, whereas evidence from heterogeneous or mixed critical-care populations remains limited. This limitation is clinically important because dogs and cats admitted to emergency and critical-care services commonly present with a broad spectrum of disorders rather than a single disease entity [2, 4, 26, 27]. In addition, serum lactate concentration has been investigated in canine and feline emergency medicine as a biomarker of tissue hypoperfusion and disease severity; however, the incremental prognostic value of lactate when combined with qSOFA in mixed populations of critically ill dogs and cats remains insufficiently characterized. Similarly, although NLR has been explored as a simple and accessible marker of systemic inflammation and clinical outcome, its additional prognostic contribution when integrated with qSOFA remains poorly understood [13, 25].

Despite increasing interest in rapid prognostic assessment tools in veterinary emergency medicine, important knowledge gaps remain regarding the comparative prognostic performance of qSOFA, lactate, SIRS, and NLR in heterogeneous populations of critically ill dogs and cats. Previous studies have largely evaluated these parameters independently or within narrowly defined disease categories, limiting their applicability to routine emergency and CCU settings where patient populations are clinically diverse. Furthermore, limited evidence is available regarding whether combining qSOFA with adjunctive biomarkers, such as lactate or NLR, provides clinically meaningful improvement in mortality prediction compared with qSOFA alone. The absence of robust comparative evidence from mixed-species and mixed-disease populations continues to hinder the development of standardized, evidence-based triage and risk-stratification protocols for veterinary critical-care.

Therefore, the aims of this study were to: (1) compare the prognostic performance of qSOFA, lactate, SIRS, and NLR for predicting mortality in critically ill dogs and cats, and (2) evaluate their usefulness as practical triage and prognostic tools for risk-stratification in critically ill dogs and cats admitted to the CCU. In addition, this study aimed to determine whether combining qSOFA with adjunctive biomarkers, particularly lactate and NLR, could improve prognostic accuracy compared with individual parameters alone. The findings of this study are expected to contribute to the development of a simple, clinically applicable, and evidence-based triage framework for small animal emergency and critical-care practice.

## MATERIALS AND METHODS

### Ethical approval

The study protocol was reviewed and approved by the Animal Care and Use Committee, Faculty of Veterinary Medicine, Chiang Mai University, Thailand (FVM-ACUC; Ref. No. S12/2566; approved on June 23, 2023). Written informed consent was obtained from all owners before enrollment of animals in the study. All procedures involving animals were performed in accordance with institutional guidelines and ethical standards for animal care, handling, and clinical investigation. The study was conducted exclusively on client-owned animals presented for routine emergency and critical-care management, and no experimental procedures beyond standard diagnostic and therapeutic interventions were performed.

### Study period and location

This prospective analytical cohort study was conducted between June and September 2023 at the Small Animal Hospital, Faculty of Veterinary Medicine, Chiang Mai University, Chiang Mai, Thailand. The study was performed in the hospital CCU, which provides emergency and intensive-care management for critically ill small animal patients.

### Study design

This study was designed and reported in accordance with the STROBE-Vet guidelines for observational studies in veterinary research. The study population consisted of critically ill dogs and cats admitted to the CCU during the study period. Initial triage assessment was performed according to previously published veterinary triage systems [28, 29], specifically using the Veterinary Triage List [30]. Animals requiring continuous intensive monitoring and advanced supportive care after stabilization were admitted to the CCU and classified as critically ill. This operational definition distinguished patients requiring sustained critical-care management from those requiring only emergency stabilization.

### Inclusion and exclusion criteria

Critically ill dogs and cats were eligible for inclusion when complete admission data were available, including signalment (breed, sex, and age), rectal temperature, heart rate, respiratory rate, systolic blood pressure, complete blood count results, and blood chemistry findings.

Animals were excluded if admission records were incomplete or if they were triaged as non-urgent and therefore not admitted to the CCU. Cases were additionally excluded when death or euthanasia occurred before CCU admission or when euthanasia was performed exclusively because of financial limitations. Furthermore, animals that responded adequately to emergency treatment and no longer required CCU admission were not enrolled in the study.

Tentative diagnoses were recorded to characterize the primary underlying disease conditions of enrolled patients. In animals presenting with multiple concurrent disorders, the condition considered to represent the principal disease process was assigned as the primary diagnosis, whereas associated complications or syndromic conditions, including sepsis, shock, or anemia, were categorized as secondary findings.

### SIRS criteria

The SIRS criteria used in this study were adapted from previously published veterinary studies [19, 31, 32]. In dogs, SIRS positivity was defined by the presence of at least two of the following abnormalities: rectal temperature <100°F or >103.5°F; heart rate >100, >120, or >160 beats/min in large-, medium-, and small-breed dogs, respectively; respiratory rate >40 breaths/min; or white blood cell count <6,000/µL, >16,000/µL, or >3% band neutrophils. Each abnormal parameter was assigned one point, and a cumulative score ≥2 was considered indicative of SIRS positivity.

In cats, SIRS criteria included rectal temperature <100°F or >103.5°F; heart rate <140 or >225 beats/min; respiratory rate >40 breaths/min; or white blood cell count <5,000 cells/µL, >19,500 cells/µL, or >5% band neutrophils. Each criterion was assigned one point, resulting in a maximum total score of four. A cumulative score ≥3 was interpreted as SIRS positivity.

### qSOFA score

The qSOFA score was determined using three clinical parameters: systolic blood pressure <100 mmHg measured by Doppler ultrasonography, respiratory rate >22 breaths/min, and altered mentation defined as a Modified Glasgow Coma Scale score <15 [2]. This threshold was used as the operational definition of altered mentation in the present study. Each parameter was assigned one point, resulting in a maximum possible score of three. A cumulative score ≥2 was considered indicative of qSOFA positivity in both dogs and cats [26, 33].

### Plasma lactate measurement

Venous blood samples (0.5-1 mL) were collected from either the cephalic or saphenous vein into lithium heparin tubes immediately after CCU admission and before the initiation of fluid resuscitation or other therapeutic interventions. Plasma lactate concentrations were analyzed using an automated chemistry analyzer (Catalyst One; IDEXX Laboratories, Inc., Westbrook, ME, USA) according to the manufacturer’s instructions.

### Data collection

All enrolled animals underwent clinical evaluation at admission, and both SIRS and qSOFA scores were assigned. Blood samples for plasma lactate analysis were collected concurrently during the admission assessment. Complete blood counts and leukocyte differential counts were analyzed using an automated hematology analyzer (BC-5300 Vet; Mindray, Shenzhen, China). When abnormal morphologic findings were flagged by the analyzer, manual blood smear evaluation was performed to verify and correct leukocyte differential counts in accordance with published recommendations that manual smear review should validate unexpected or flagged automated hematologic results [34].

Animals were monitored throughout hospitalization and followed for 21 days after admission. Clinical outcome was classified as survival if the animal remained alive at day 21 post-admission. Non-survival was defined as death or euthanasia, excluding euthanasia performed solely because of financial constraints, occurring within the 21-day observation period. Animals remaining hospitalized at day 21 were classified as survivors for the primary outcome analysis.

### Statistical analysis

Data were analyzed on a complete-case basis for each comparison. The primary outcome variable was mortality status, categorized as survivor or non-survivor. Continuous variables, including age and lactate concentration, were expressed as medians and ranges, whereas categorical variables, including species and sex, were summarized as percentages.

Mortality discrimination was evaluated using receiver operating characteristic (ROC) curve analysis and area under the curve (AUC) values for three predictive models: (1) qSOFA in the full study cohort; (2) lactate concentration in the subset with available lactate measurements; and (3) a combined qSOFA-lactate model constructed using logistic regression in the intersection subset. For each model, 95% confidence intervals were calculated using the DeLong method. Pairwise comparisons between AUC values were performed using DeLong’s test on the shared intersection subset.

Clinically practical triage thresholds were evaluated using four predefined approaches: (1) qSOFA ≥2; (2) a qSOFA threshold selected using Youden’s index (J = sensitivity + specificity − 1); (3) a lactate threshold (L*) selected using Youden’s index within the lactate-measured subset, together with clinically relevant lactate cut-offs of ≥2.0 and ≥4.0 mmol/L [1]; and (4) an escalation rule defined as qSOFA ≥2 or lactate ≥L*. For each rule, sensitivity, specificity, and complete classification tables were calculated. Uncertainty estimates for sensitivity and specificity were determined using a non-parametric percentile bootstrap approach and expressed as 95% confidence intervals.

Youden-derived thresholds were generated from ROC curves using the full cohort for qSOFA analyses and the lactate-measured subset for lactate analyses. Additional thresholds for SIRS and NLR were also evaluated. To maximize specificity, a cutoff score of SIRS = 4 was selected for performance analysis. The NLR cutoff value was derived from ROC analysis using Youden’s index. Escalation rules combining qSOFA with SIRS (qSOFA ≥2 or SIRS = 4) and qSOFA with NLR (qSOFA ≥2 or NLR ≥0.04) were additionally assessed.

All statistical analyses and figure generation were performed using Python software version 3.11.2 (Python Software Foundation, Wilmington, DE, USA). AUC confidence intervals and pairwise ROC comparisons were computed using a DeLong implementation. All statistical tests were two-sided, and statistical significance was defined as p < 0.05. Results are presented as estimates with 95% confidence intervals without multiplicity adjustment.

## RESULTS

### Baseline demographic and clinical characteristics

A total of 76 emergency cases were enrolled in the study, comprising 39 dogs (51.3%) and 37 cats (48.7%). The median age was 9 years (range: 0.25-16 years) in dogs and 3 years (range: 0.25-15 years) in cats. Animals younger than 1 year of age were expressed in months, where applicable (e.g., 3 months = 0.25 years).

Among dogs, 22/39 (56.4%) were male, including 10 intact males (45.5%) and 12 castrated males (54.5%), whereas 17/39 (43.6%) were female, including 9 intact females (52.9%) and 8 spayed females (47.1%). In cats, 26/37 (70.3%) were male, including 18 intact males (69.2%) and 8 castrated males (30.8%), while 11/37 (29.7%) were female, including 8 intact females (72.7%) and 3 spayed females (27.3%).

Mixed-breed dogs represented the largest canine subgroup (13/39, 33.3%), followed by Chihuahua (6/39, 15.4%), Poodle (4/39, 10.3%), and Pomeranian (4/39, 10.3%), whereas the remaining breeds collectively accounted for 12/39 (30.7%) of cases. Among cats, Domestic Short Hair cats represented the predominant breed (33/37, 89.2%), followed by Persian cats (2/37, 5.4%), whereas Scottish Fold and Siamese cats each accounted for 1/37 (2.7%).

The overall 21-day survival rate was 47.4% (36/76), including 51.3% (20/39) in dogs and 43.2% (16/37) in cats.

### Distribution of lactate, qSOFA, NLR, and SIRS values

**Figure 1:**
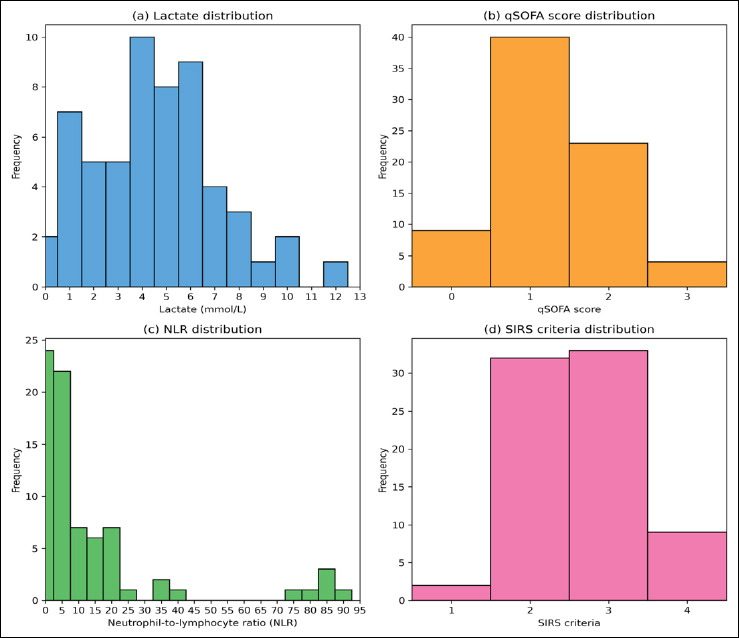
Frequency distributions of clinical parameters in hospitalized dogs and cats. (a) Blood lactate concentration, (b) neutrophil-to-lymphocyte ratio (NLR), (c) quick Sequential Organ Failure Assessment (qSOFA) score, and (d) Systemic Inflammatory Response Syndrome (SIRS) score among dogs and cats admitted to the critical-care unit

As illustrated in Figure 1a, plasma lactate concentrations were available for 57 of the 76 enrolled animals, with a median concentration of 4.80 mmol/L (range: 0.83-12.00 mmol/L). Figure 1b demonstrates the distribution of qSOFA scores among all enrolled cases, with a median score of 1.00 (range: 0-3). Figure 1c presents the distribution of NLR values, which showed a median value of 8.35 (range: 0-94), whereas Figure 1d illustrates the distribution of SIRS scores, with a median score of 3 (range: 1-4).

### Distribution of sepsis, sepsis-suspected, and non-sepsis cases

For descriptive categorization, cases were classified as sepsis when suspected or confirmed infection was present together with fulfillment of the SIRS criteria [19]. As presented in Table 1, 25/76 (32.9%) animals were categorized as sepsis cases, 23/76 (30.3%) as sepsis-suspected cases, and 28/76 (36.8%) as non-sepsis cases.

**Table 1:** Classification of patients into sepsis, sepsis-suspected, and non-sepsis groups and the primary diagnoses.

Species	Group	n	%	Primary diagnoses
Dog	Sepsis	13	33.3	UTI (n = 3); pyometra (n = 3); gastrointestinal perforation (n = 2); pneumonia (n = 2); wound infection/bite wound (n = 2); discospondylitis (n = 1)
	Sepsis-suspected	12	30.8	CPV (n = 4); UTI (n = 3); wound infection/open fracture (n = 2); pyometra (n = 2); gastrointestinal perforation (n = 1)
	Non-sepsis	14	35.9	Pancreatitis (n = 4); CKD (n = 3); CHF (n = 2); intoxication (n = 2); portosystemic shunt (n = 1); bone fracture (n = 1); idiopathic epilepsy (n = 1)
Cat	Sepsis	12	32.5	Septic peritonitis (n = 4); UTI (n = 3); pyothorax (n = 2); hepatic abscess (n = 1); wound infection/bite wound (n = 1); pyometra (n = 1)
	Sepsis-suspected	11	29.7	Feline panleukopenia virus infection (n = 3); wound infection/bite wound (n = 2); UTI (n = 2); pyometra (n = 2); gastrointestinal perforation (n = 1); wound infection/open fracture (n = 1)
	Non-sepsis	14	37.8	FeLV-associated disease (n = 4); blunt trauma (n = 2); CHF (n = 2); FeLV/FIV co-infection (n = 2); intoxication (n = 1); acute kidney injury (n = 1); feline immunodeficiency virus infection (n = 1); brain trauma (n = 1)

Group criteria: Sepsis = infection confirmed by hemoculture together with fulfillment of SIRS criteria; Sepsis-suspected = suspected infection with negative hemoculture (no growth) or infection not definitively confirmed together with fulfillment of SIRS criteria; Non-sepsis = absence of evidence of infection or absence of fulfillment of SIRS criteria. UTI = urinary tract infection, CPV = canine parvovirus, CKD = chronic kidney disease, CHF = congestive heart failure, FeLV = feline leukemia virus, FIV = feline immunodeficiency virus, CCU = critical-care unit, SIRS = systemic inflammatory response syndrome.

In dogs, sepsis was most frequently associated with urinary tract infection (UTI) and pyometra, whereas sepsis-suspected cases were primarily associated with canine parvovirus (CPV) infection and UTI. Non-sepsis conditions in dogs included pancreatitis, chronic kidney disease (CKD), congestive heart failure (CHF), intoxication, portosystemic shunt, bone fracture, and idiopathic epilepsy.

In cats, sepsis was most commonly associated with septic peritonitis, UTI, and pyothorax. Sepsis-suspected cases included feline panleukopenia virus infection, wound infection/bite wounds, UTI, pyometra, gastrointestinal perforation, and wound infection/open fracture. Non-sepsis conditions included feline leukemia virus-associated disease, blunt trauma, CHF, feline leukemia virus/feline immunodeficiency virus co-infection, intoxication, acute kidney injury, feline immunodeficiency virus infection, and brain trauma.

### ROC analysis and pairwise DeLong comparisons

Pairwise DeLong comparisons demonstrated that qSOFA showed significantly superior prognostic performance compared with lactate ≥2.0 mmol/L (p = 0.004), lactate ≥4.0 mmol/L (p = 0.005), and qSOFA + lactate ≥2.0 mmol/L (p = 0.007). However, the comparison between qSOFA and qSOFA + lactate ≥4.0 mmol/L did not reach statistical significance (*p* = 0.064) (Table 2).

**Table 2:** Pairwise DeLong comparisons of area under the receiver operating characteristic curve between predictive models in the pooled cohort (n = 76).

Comparison	AUC Model 1	AUC Model 2	z-value	p-value
qSOFA versus lactate ≥1.46 mmol/L	0.72	0.47	1.28	0.201
qSOFA versus lactate ≥2.0 mmol/L	0.72	0.47	-2.89	0.004
qSOFA versus lactate ≥4.0 mmol/L	0.72	0.45	-2.84	0.005
qSOFA versus qSOFA + lactate ≥1.46 mmol/L	0.72	0.75	-0.30	0.761
qSOFA versus qSOFA + lactate ≥2.0 mmol/L	0.72	0.55	-2.69	0.007
qSOFA versus qSOFA + lactate ≥4.0 mmol/L	0.72	0.60	-1.86	0.064
qSOFA versus SIRS	0.72	0.48	2.85	0.004
qSOFA versus qSOFA + SIRS	0.72	0.72	0.04	0.968
SIRS versus qSOFA + SIRS	0.48	0.72	-2.30	0.021
qSOFA versus NLR	0.72	0.38	3.34	<0.001
qSOFA versus qSOFA + NLR	0.72	0.73	-0.09	0.932
NLR versus qSOFA + NLR	0.38	0.73	-2.86	0.004

qSOFA = Quick Sequential Organ Failure Assessment score, SIRS = Systemic Inflammatory Response Syndrome, NLR = Neutrophil-to-lymphocyte ratio.

**Figure 2:**
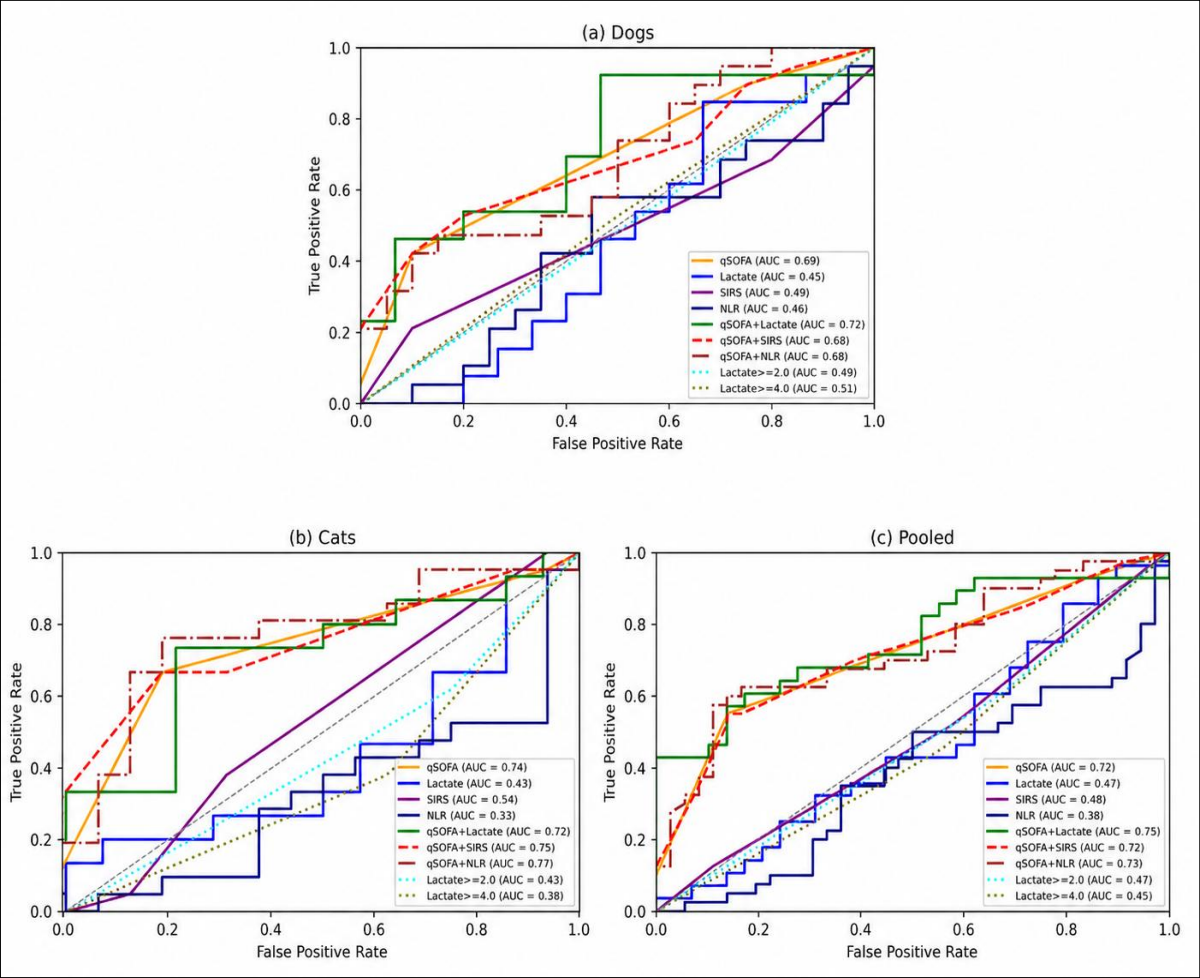
Receiver operating characteristic curves for prediction of 21-day mortality in critically ill dogs and cats. (a) Dogs (n = 39), (b) cats (n = 37), and (c) pooled cohort (n = 76). Curves are shown for quick Sequential Organ Failure Assessment (qSOFA), lactate, Systemic Inflammatory Response Syndrome (SIRS), neutrophil-to-lymphocyte ratio (NLR), combined models, and lactate threshold models. Lactate-based comparisons were performed using the lactate-measured subset (n = 57), whereas non-lactate comparisons used the full cohort (n = 76). AUC = area under the receiver operating characteristic curve.

As illustrated in Figure 2, qSOFA scores were significantly higher among non-survivors. ROC analysis demonstrated that qSOFA achieved an AUC of 0.72 (95% confidence interval [CI]: 0.61-0.82), indicating good discriminatory performance for mortality prediction. Although the combined qSOFA+lactate model produced a slightly greater AUC of 0.75 (95% CI: 0.62-0.88) within the lactate subset, this increase was not statistically significant compared with qSOFA alone (p = 0.761).

### Threshold performance of candidate CCU triage rules

As shown in Table 3, qSOFA ≥2 demonstrated a sensitivity of 0.55 and specificity of 0.86 in the overall study cohort (n = 76). Within the lactate subset (n = 57), the Youden-optimal lactate threshold was ≥1.46 mmol/L, yielding a sensitivity of 0.96 and specificity of 0.10. Increasing the lactate cutoff to ≥2.0 mmol/L decreased sensitivity to 0.86 while increasing specificity to 0.17. At a threshold of ≥4.0 mmol/L, sensitivity further decreased to 0.64, whereas specificity improved to 0.31.

**Table 3:** Threshold performance for candidate critical-care unit triage rules in the pooled cohort (n = 76).

Threshold (n)	Sensitivity (95% CI)	Specificity (95% CI)	TP	FP	TN	FN
qSOFA ≥2 (76)	0.55 (0.39–0.71)	0.86 (0.74–0.97)	22	5	31	18
Lactate ≥1.46 mmol/L (57)	0.96 (0.88–1.00)	0.10 (0.00–0.22)	27	26	3	1
Lactate ≥2.0 mmol/L (57)	0.86 (0.69–0.94)	0.17 (0.08–0.35)	24	24	5	4
Lactate ≥4.0 mmol/L (57)	0.64 (0.46–0.79)	0.31 (0.17–0.49)	18	20	9	10
qSOFA ≥2 or lactate ≥1.46 mmol/L (57)	1.00 (1.00–1.00)	0.10 (0.00–0.22)	28	26	3	0
qSOFA ≥2 or lactate ≥2.0 mmol/L (57)	0.93 (0.78–0.98)	0.17 (0.08–0.35)	26	24	5	2
qSOFA ≥2 or lactate ≥4.0 mmol/L (57)	0.89 (0.73–0.96)	0.31 (0.17–0.49)	25	20	9	3
SIRS = 4 (76)	0.12 (0.03–0.24)	0.89 (0.77–0.98)	5	4	32	35
qSOFA ≥2 or SIRS = 4 (76)	0.62 (0.47–0.77)	0.75 (0.59–0.89)	25	9	27	15
NLR ≥0.04 (76)	0.97 (0.92–1.00)	0.03 (0.00–0.09)	39	35	1	1
NLR ≥5 (76)	0.63 (0.46–0.77)	0.25 (0.12–0.42)	25	27	9	15
qSOFA ≥2 or NLR ≥0.04 (76)	1.00 (1.00–1.00)	0.00 (0.00–0.00)	40	36	0	0
qSOFA ≥2 or NLR ≥5 (76)	0.93 (0.80–0.98)	0.17 (0.06–0.33)	37	30	6	3

The qSOFA analysis converged on the same integer threshold (≥2), producing identical operating characteristics within this dataset. TP = true positives, FP = false positives, TN = true negatives, FN = false negatives, CI = confidence interval. qSOFA = Quick Sequential Organ Failure Assessment score, SIRS = Systemic Inflammatory Response Syndrome, NLR = Neutrophil-to-lymphocyte ratio.

Combining qSOFA ≥2 with lactate ≥1.46 mmol/L resulted in a sensitivity of 1.00 but a specificity of only 0.10. Similarly, escalation rules incorporating lactate thresholds of ≥2.0 mmol/L and ≥4.0 mmol/L yielded sensitivities of 0.93 and 0.89, respectively, with corresponding specificities of 0.17 and 0.31.

### Diagnostic performance of SIRS and NLR thresholds

The SIRS cutoff value of 4 demonstrated high specificity (89%) but poor sensitivity (12%), with 35/40 non-survivors exhibiting SIRS scores below this threshold. Conversely, a low NLR threshold identified nearly all non-survivors (97% sensitivity) but incorrectly classified nearly all survivors (3% specificity), resulting in the escalation rule (qSOFA ≥2 or NLR ≥0.04) categorizing all animals as positive.

At a higher NLR threshold (>5), sensitivity for detecting non-survivors decreased to 62.5%, whereas specificity remained low at 25.0%. Combining qSOFA ≥2 with NLR >5 increased sensitivity to 92.5% but further reduced specificity to 16.7%. Among all evaluated triage rules, qSOFA ≥2 demonstrated the most balanced diagnostic performance, with a sensitivity of 0.55 and specificity of 0.86. The addition of SIRS = 4 as an escalation criterion modestly improved sensitivity but reduced specificity.

## DISCUSSION

### Prognostic performance of qSOFA and lactate

In this mixed population of critically ill dogs and cats, qSOFA demonstrated fair discrimination for 21-day mortality prediction, with an AUC of approximately 0.72, whereas lactate alone showed poor prognostic performance. These findings are consistent with previous studies in dogs with pyometra and surgically treated sepsis, in which qSOFA ≥2 was associated with higher mortality and longer hospitalization [26, 29]. The present findings support qSOFA as a clinically relevant indicator of physiologic derangement in heterogeneous populations that include sepsis, sepsis-suspected, and non-sepsis cases.

Species-specific analysis showed that qSOFA ≥2 had an AUC of 0.69 in dogs, with sensitivity of 0.42 and specificity of 0.90, and an AUC of 0.74 in cats, with sensitivity of 0.67 and specificity of 0.81. These results suggest slightly stronger discriminative performance in cats than in dogs. Therefore, qSOFA performance may vary according to species, disease distribution, and clinical case mix rather than being uniformly applicable across all critically ill small animals.

In contrast, lactate alone showed limited prognostic value in this cohort, with an AUC of approximately 0.47, and did not significantly improve qSOFA when incorporated into a combined model. This differs from human emergency medicine studies, in which adding lactate ≥2.0 mmol/L to qSOFA improved sensitivity for adverse outcomes from 0.47 to 0.65 [35]. Although veterinary studies have reported similar trends, the incremental benefit has generally been modest. In a cohort of 267 dogs admitted to a CCU, qSOFA combined with lactate achieved AUCs of 0.62-0.64, whereas qSOFA alone showed poor discrimination, with an AUC of approximately 0.60, indicating only a slight improvement in predictive ability [1]. In the present study, the combined qSOFA-lactate model resulted in only a small increase in AUC, from 0.72 to 0.75, and pairwise DeLong testing showed no significant improvement. These discrepancies may reflect species-related differences, heterogeneous disease processes, differences in lactate thresholds, and variation in the timing of lactate measurement. In particular, very low lactate cut-offs may reflect physiologic stress but substantially reduce specificity.

### Prognostic value of SIRS and NLR

In addition to qSOFA and lactate, two additional candidate predictors, SIRS and NLR, were evaluated. Both variables performed poorly as standalone mortality classifiers, with an AUC of approximately 0.48 for SIRS and 0.38 for NLR. When either marker was added to qSOFA in logistic models, the combined AUC remained approximately 0.72, indicating no meaningful improvement over qSOFA alone.

At the selected cut-offs, SIRS = 4 was highly specific, with specificity of 0.89, but poorly sensitive, with sensitivity of 0.12. Conversely, NLR ≥0.04 was highly sensitive, with sensitivity of 0.97, but lacked specificity, with specificity of 0.03. Because this threshold was far below typical reference ranges and effectively classified nearly all animals as high-risk, NLR did not provide a clinically useful triage cutoff in this study. An escalation rule using qSOFA ≥2 or SIRS = 4 increased sensitivity to 0.62 but reduced specificity to 0.75. In contrast, combining qSOFA with the low NLR threshold classified all animals as positive. These findings indicate that SIRS and NLR added limited prognostic value beyond qSOFA in this cohort.

The weak prognostic performance of SIRS may have been influenced by the study inclusion criteria. Enrollment was restricted to critically ill animals, many of which already fulfilled inflammatory criteria at admission, resulting in a population enriched for systemic inflammation at baseline. This reduced the available clinical spectrum and likely diminished the ability of SIRS to discriminate survivors from non-survivors. This selection may also have influenced the observed performance of qSOFA, NLR, and lactate, because all markers were assessed within a preselected high-acuity population rather than across the full spectrum of emergency presentations.

Although a very low NLR cutoff identified almost all non-survivors, a higher threshold of >7 was also evaluated because it may better represent systemic inflammation [36]. This cutoff showed only modest sensitivity and very low specificity, resulting in poor discriminative ability when used alone. When combined with qSOFA, using qSOFA ≥2 or NLR >5, sensitivity increased substantially, but specificity declined markedly. In a prospective study of critically ill dogs, NLR showed only weak correlations with illness severity and did not reliably predict survival [13]. Similarly, in cats with blunt trauma, NLR increased with injury severity, reflected by higher Animal Trauma Triage scores, but was not associated with hospitalization duration, intensive-care needs, or mortality [37].

In healthy dogs, published reference intervals for NLR range from approximately 1.3 to 7.1 [36]. Prognostic studies have also reported cut-offs around 5 for differentiating survivors from non-survivors; for example, a study of canine mammary tumors identified NLR = 5 as the threshold, with 72.7% sensitivity and 74.6% specificity [38]. In contrast, stronger performance has been reported in disease-specific contexts, such as dogs with acute CHF, in which an NLR cutoff of approximately 8.2 achieved an AUC of 0.91 for short-term outcomes [39]. In humans, a meta-analysis of adult sepsis reported a pooled AUC of 0.80, sensitivity of 0.64, and specificity of 0.79, whereas meta-regression identified study design and timing of NLR measurement as key sources of heterogeneity [40]. Beyond sepsis, a large geriatric inpatient cohort identified an optimal NLR cutoff of 7.95, with an AUC of 0.707, and a general population study in humans linked higher NLR quartiles with increased all-cause and cause-specific mortality, emphasizing the prognostic signal of NLR while highlighting threshold variability across clinical contexts [41].

### Threshold performance and clinical interpretation

Threshold analyses help translate predictive performance into clinically applicable decision-making. In this study of CCU-admitted animals, qSOFA ≥2 achieved a sensitivity of 0.55 and specificity of 0.86 for 21-day mortality, supporting its use as a practical CCU triage and risk-stratification rule. Lactate thresholds performed poorly as standalone predictors. At the Youden-derived cutoff of 1.46 mmol/L, lactate sensitivity was 0.96, but specificity was only 0.10. Increasing the lactate cutoff to ≥2.0 mmol/L reduced sensitivity to 0.86 while improving specificity to 0.17. At ≥4.0 mmol/L, sensitivity decreased to 0.64, whereas specificity increased to 0.31.

Escalation rules that classified animals as positive when qSOFA ≥2 or lactate ≥2.0 or ≥4.0 mmol/L improved sensitivity to 0.93 and 0.89, respectively, but this occurred at the expense of specificity, which remained low at 0.17 and 0.31, respectively. These results reflect classification of survivors versus non-survivors within a CCU population rather than selection for CCU admission. Consequently, lactate appears better suited as an adjunctive marker to prompt closer monitoring and reassessment rather than as a primary admission criterion.

### Comparative diagnostic accuracy in veterinary and human studies

Overall, qSOFA in the present veterinary study demonstrated fair predictive ability for mortality, whereas SIRS, NLR, and lactate each showed little to no discriminative power as standalone predictors. In contrast, human studies have reported moderate accuracy for both qSOFA and SIRS in predicting death. For example, in a 28-day sepsis study, qSOFA achieved an AUC of 0.67 compared with 0.62 for SIRS [42], and the original Sepsis-3 analyses reported an AUC of 0.66 for qSOFA compared with 0.64 for SIRS [43]. Therefore, qSOFA performance in animals appears broadly comparable to that reported in humans, whereas SIRS was markedly less predictive in the present study.

Nearly all critically ill patients in this cohort fulfilled SIRS positivity by inclusion or clinical presentation, resulting in near-random mortality prediction by SIRS. This contrasts with human data, where SIRS, despite its well-recognized limitations, retains some prognostic value for mortality prediction. NLR was also uninformative as a standalone marker in this study, whereas human studies have reported stronger associations between elevated NLR and increased all-cause mortality [41, 44]. A similar association was not observed in the present veterinary dataset.

Blood lactate also showed limited utility as a standalone predictor in this study. A very low threshold of approximately 1.5 mmol/L was required to achieve high sensitivity and capture nearly all deaths, but this resulted in very poor specificity. In contrast, lactate is a well-established mortality marker in human sepsis. In one study, an initial lactate concentration >3 mmol/L had a sensitivity of 0.65 for in-hospital death [24]. Furthermore, adding lactate elevation to qSOFA has been shown to substantially improve identification of high-risk patients in human studies [33, 45-47]. In the present veterinary population, adding lactate did not meaningfully improve predictive accuracy. This difference may be explained by the clinical heterogeneity of the study population, inclusion of disorders not primarily driven by systemic hypoperfusion, single-time-point lactate assessment at admission, incomplete lactate data, reduced statistical power, and the very low Youden-derived threshold that produced high sensitivity but extremely poor specificity.

### Clinical implications

In this study, qSOFA ≥2 identified more than half of the non-survivors, with sensitivity of 0.55 and high specificity of 0.86, supporting its role as a practical CCU triage trigger and adjunctive risk-stratification tool in critically ill dogs and cats. Lactate, although highly sensitive at lower thresholds, lacked sufficient specificity for independent triage decisions and may be more useful as an adjunctive marker for enhanced monitoring. Therefore, elevated lactate concentrations should be interpreted as non-specific early warning signals rather than standalone admission criteria.

SIRS criteria were positive in many critically ill patients, and NLR showed minimal predictive value, limiting their usefulness for mortality prediction in this population. Based on these findings, qSOFA ≥2 may help identify high-risk cases at triage, whereas lactate may serve as a secondary marker to prompt closer monitoring and repeated clinical reassessment. However, qSOFA should be interpreted together with clinical judgment, patient history, serial examination, and ongoing reassessment rather than used as a standalone triage criterion.

### Limitations and future directions

This study was limited by its single-center design, modest sample size, and incomplete lactate data. External validity may be affected by the local epidemiology, referral characteristics, and case distribution of the study hospital; therefore, the findings should be generalized cautiously to primary-care, non-referral, or geographically distinct veterinary populations. Lactate measurements were available in only 57 of 76 animals, reducing statistical power. Because lactate sampling depended on feasibility during emergency presentation, selection bias related to missing lactate data cannot be excluded. In addition, potentially relevant variables, including comorbidities, body weight, treatment interventions, and serial measurements of qSOFA or lactate, were not evaluated.

The clinical heterogeneity of the study population, which included both dogs and cats with a wide range of diseases, may further limit the generalizability of the findings. Moreover, enrollment was restricted to critically ill animals, resulting in enrichment for high-acuity cases at baseline and possible spectrum bias. Consequently, these findings may not fully reflect the performance of qSOFA, lactate, SIRS, and NLR in broader emergency-room populations that include animals with milder disease severity.

Prospective multicenter studies are needed to validate qSOFA thresholds across different veterinary hospitals, species, and disease categories. Future research should also evaluate serial qSOFA and lactate measurements, additional biomarkers, dynamic scoring approaches, and the potential application of machine-learning models for veterinary critical-care triage and mortality prediction.

## CONCLUSION

In this mixed population of critically ill dogs and cats, qSOFA ≥2 demonstrated fair discrimination for 21-day mortality and showed the most balanced overall performance among the evaluated prognostic indicators. qSOFA achieved an AUC of 0.72, with sensitivity of 0.55 and specificity of 0.86, supporting its value as a simple and clinically practical risk-stratification tool in small animal critical-care. In contrast, lactate alone showed limited prognostic accuracy, and although lower lactate thresholds provided high sensitivity, they were associated with poor specificity and a high false-positive rate. Adding lactate, SIRS, or NLR to qSOFA did not meaningfully improve mortality prediction in this cohort.

The practical implication of these findings is that qSOFA may help clinicians rapidly identify dogs and cats at higher risk of death during CCU admission, particularly when used alongside clinical judgment and repeated patient reassessment. Lactate may be useful as an adjunctive escalation marker to prompt closer monitoring, but it should not be used as a standalone admission or mortality prediction criterion. SIRS and NLR showed limited utility for prognostic decision-making in this heterogeneous critically ill population.

A major strength of this study is its prospective design and direct comparison of multiple clinically accessible prognostic indicators in both dogs and cats admitted to a CCU. However, the single-center setting, modest sample size, incomplete lactate data, and clinically heterogeneous case population may limit generalizability. Future multicenter studies with larger cohorts should validate qSOFA thresholds, assess serial qSOFA and lactate measurements, and explore additional biomarkers or dynamic prediction models for veterinary critical-care triage.

Overall, qSOFA may serve as a simple, rapid, and practical adjunctive tool for mortality risk-stratification in critically ill dogs and cats. Its greatest value is likely achieved when integrated with clinical judgment, serial monitoring, and other diagnostic information rather than applied as an isolated prognostic criterion.

## DATA AVAILABILITY

The supplementary data can be made available from the corresponding author upon request.

## AUTHORS’ CONTRIBUTIONS

BB and PC: Study conception and design, data analysis and interpretation, and drafted and revised the manuscript. BB, PC, TC, JJ, and CS: Sample collection, experiments and data analysis and interpretation. BB: Statistical analysis. All authors have read and approved the final version of the manuscript.
